# The Doctor vs. the Minister

**Published:** 1892-05

**Authors:** 


### THE DOCTOR VS. THE MINISTER.

The question which seems now to be agitating the medical press is the relations existing between the medical man and the minister of the gospel, and the attitude of each to the other.

All the discussion upon the matter seems to have been brought about by the presentation of the bill of a celebrated Brooklyn physician against the estate of a Catholic priest for services rendered, which the heirs protested upon the grounds that it was not customary with the profession to make charges for medical services rendered such persons.

Taking a retrospective view of the field, from time almost immemorial the members of the medical profession have had no more potent enemy than has existed in the representatives of the sister profession. No class of people can be found who are more ready to give their sanction and endorsement to a quack measure or a patent nostrum than they, whose signature to a testimonial glowing with enthusiasm and unreserved commendation carries with it so much weight upon the mind of the laity.

The quack nostrum vendor and the patent medicine manufacturer realizes to its fullest extent the truthfulness of this latter remark, and the natural outcome of such a status of affairs is that the secular press of to-day is teeming with testimonials of the wonderful and miraculous powers possessed by some certain patent medicine in curing some self-diagnosed disease which, in all probability, never existed at all, and attached to them are the signatures of Rev. Mr. ———, and Dr. ———, pastor of ——— church, and Bishop ———, etc.

These men, who are presumed to be the shepherds of their flocks, who are supposed to be the friends of weak and wayward mankind, and who claim to be men of learning and scientific attainments, are to-day completely ignoring legitimate medicine based upon centuries of diligent research and scientific investigation, and thus pouring dollars into the coffers of the sharp and keen-witted manufacturers of these goods; and "'tis true—and pity 'tis 'tis true"—*in some cases actually owning stock in the manufacture of the very nostrum they are so assiduous in advertising.*

Now, let one of these be stricken with some serious illness—one who has been most ardent in his endeavors to proclaim to the world (prompted, of course, by a deep and burning love for poor suffering humanity) the merits of some nostrum which he has found a panacea for every ill to which human flesh is heir. Does he send for the learned "doctor" whose name this preparation bears, or for a bottle of this "wonderful discovery?" We challenge the world for the citation of a single instance. The physician, the humble slave at the throne of Æsculapius, the martyr to his countrymen, the elegant gentleman and professional scholar, and the man whom he has made so desperate an attempt to overthrow, is the one who is summoned, and yet, he not only receives, but expects these services free of all charge.

Section 3, Article I, Chapter III of the code says: "There is no profession by the members of which eleemosynary services are more liberally dispensed than the medical, but justice requires that some limit should be placed to the performance of such good offices. Poverty, professional brotherhood and certain of the public duties referred to in the first section of this article, should always be recognized as presenting valid claims for gratuitous services; but neither * * * * * * *nor any profession or occupation can be admitted to possess such privilege.*"

In what does their claim for exemption from fees exist, and why should they, because of their calling simply, demand and receive attendance upon themselves and their families gratuitously?

